# Evaluation of extra‐corporeal membrane oxygenator cannulae in pulsatile and non‐pulsatile pediatric mock circuits

**DOI:** 10.1111/aor.14897

**Published:** 2024-10-28

**Authors:** Lorenzo Ferrari, Maris Bartkevics, Hansjörg Jenni, Alexander Kadner, Matthias Siepe, Dominik Obrist

**Affiliations:** ^1^ ARTORG Center for Biomedical Engineering Research University of Bern Bern Switzerland; ^2^ Department of Cardiac Surgery, Inselspital Bern University Hospital, University of Bern Bern Switzerland

**Keywords:** cannulae, compliance, extracorporeal circulation, hemodynamics, mock circulation, pediatric, pulsatile flow

## Abstract

**Background:**

This study evaluated the hemodynamic performance of arterial and venous cannulae in a compliant pediatric extracorporeal membrane oxygenation (ECMO) mock circuit in pulsatile and non‐pulsatile flow conditions.

**Methods:**

The ECMO setup consisted of an oxygenator, diagonal pump, and standardized‐length arterial/venous tubing with pressure transducers. A validated left‐heart mock loop was adapted to simulate pediatric conditions. The pulsatile flow was driven by a computer‐controlled piston pump set at 120 bpm. A roller pump was used for non‐pulsatile conditions. The circuit was primed with 40% glycerol‐based solution. The cardiac output was set to 1 L/min and the aortic pressure to 40–50 mmHg. Four arterial cannulae (8Fr, 10Fr, 12Fr, 14Fr) and five venous cannulae (12Fr, 14Fr, 16Fr, 18Fr, 20Fr) (Medtronic, Inc., Minneapolis, MN, USA) were tested at increasing flow rate in 12 combinations.

**Results:**

The pulsatile condition required lower ECMO pump speeds for all cannulae combinations at a given flow rate, inducing a significantly smaller increase of flow in the mock loop. Under non‐pulsatile conditions, the aortic and arterial pressures in the cannulae were higher (*p* < 0.01) while no significant differences in pressure drop and pressure‐flow characteristics (M‐number) were observed. The total hemodynamic energy was higher in case of non‐pulsatile flow (*p* < 0.01).

**Conclusion:**

Under non‐pulsatile conditions, the system was characterized by overall higher pressures, resulting in higher support to the patient. The consequent increase of potential energy compensates for increases of kinetic energy, leading to a higher total hemodynamic energy. Pressure gradients and *M number* are independent of the testing conditions. Pulsatile testing conditions led to more physiological testing conditions, and it is recommended for ECMO testing.

## Graphical abstract

This study aims to evaluate ECMO cannulae in pulsatile and non‐pulsatile pediatric mock circuits. Under non‐pulsatile conditions, the system was characterized by overall higher pressures, resulting in lower support to the patient and higher total hemodynamic energy. Pressure gradients and *M number* were not affected by the testing conditions.

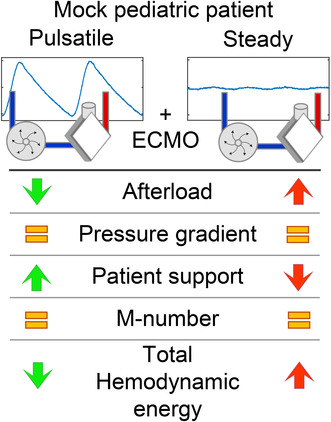

## 1 INTRODUCTION

The clinical use of extracorporeal membrane oxygenation (ECMO) support has doubled in the last decade, and over 35 000 ECMO treatments were reported for pediatric patients in 2022 worldwide.^1^ Compared to ECMO in adult patients, pediatric ECMO has different indications, parameter settings, and implantation sites. Pediatric patients are very heterogeneous, showing significant variations in weight and vessel dimensions. Thus, selecting an appropriate cannula size and combination is an important clinical decision which may lead to adverse effects such as hemolysis, high resistance, and hemodilution.^2^, ^3^ The cannula needs to be narrow enough to be inserted in the patient vessel without obstructing the flow, but wide enough to provide adequate metabolic support. For the venous cannula, only relatively low drainage pressures are possible to prevent the collapse of the vessel.^4^ Manufacturers present pressure‐flow diagrams to support the clinical selection of the most suitable cannula dimension. However, the simplicity of the testing conditions (e.g., water as testing fluid, steady flow conditions) could lead to misleading results. Some studies introduced blood or blood analogs as testing fluids to better model the clinical situation. In 2011, Qiu et al. tested different Venous–Arterial (VA) cannulae in a simulated neonatal extracorporeal life support (ECLS) circuit primed with blood.^5^ In 2016, Wang et al. used a 40% hematocrit Ringer's lactate solution to evaluate two arterial cannulae in a simulated Neonatal Cardiopulmonary Bypass Circuit.^6^ Three years later, the same authors investigated the impact of cannula size and line length on venous line pressure in pediatric ECLS circuits.^6^ In these experiments, the patient was modeled by a static reservoir of 300‐500 mL. The venous pressure was maintained constant while the arterial pressure was imposed with a Hoffman clamp. The results quantified the significant influence of the tubing length on the venous line pressure. Moreover, the impact of the arterial cannula on the flow rate was higher than for the venous cannula. Concerning pulsatile ECLS, several studies have been conducted in the past years. In addition to the above‐mentioned contributions to cannulae evaluation,^3^, ^7^ pulsatile ECLS systems were extensively studied.^8^, ^9^, ^10^, ^11^, ^12^, ^13^, ^14^, ^15^ Moreover, an overview of recent studies on pulsatile systems for blood delivery in ECLS can be found in the work of Kanagarajan et al.^16^ However, the authors note that the present study does not aim at investigating pulsatile pumping systems in ECLS. Instead, the present study evaluates how the systemic pulsatility typically observed in patients can affect the evaluation of ECMO components. Pulsatile flow typically carries higher kinetic energy levels compared to non‐pulsatile flow at the same flow rate and mean pressure.^17^ With this focus, Wickramarachchi et al. recently published a study evaluating different cannula sizes and Venous–Arterial (VA) ECMO flow rates for different adult patients in a realistic hydraulic loop mimicking systemic resistance and heart rate.^18^

In this study, we designed a mock loop mimicking systemic resistance and compliance of a toddler with preserved systemic pulsatility. The mock loop was paired with a complete ECMO circuit and used to test different combinations of arterial and venous cannulae with and without patient pulsatility.

## 2 MATERIALS AND METHODS

### 2.1 Pseudo‐patient

**FIGURE 1:**
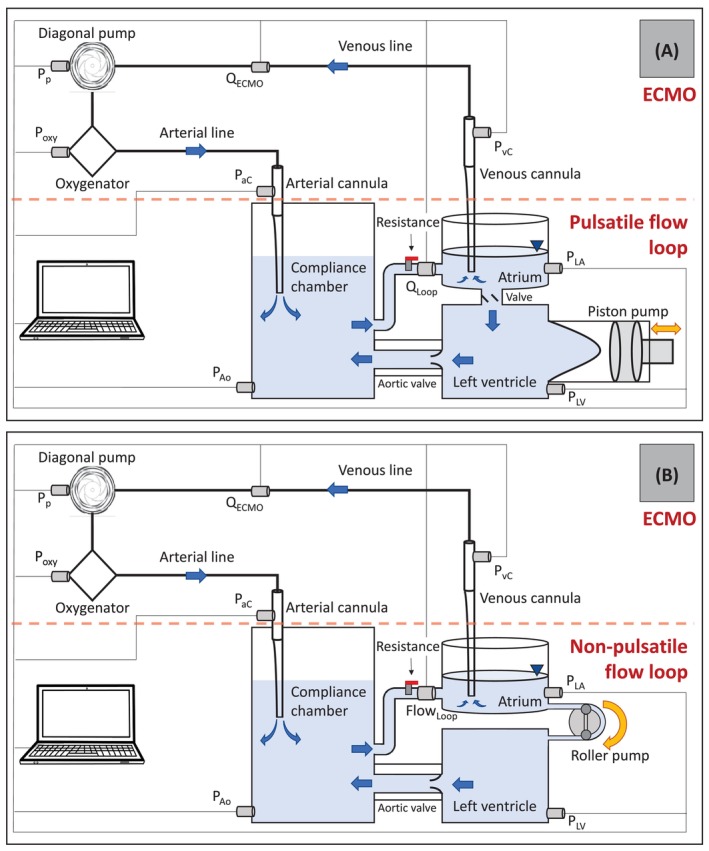
Experimental setup: Pulsatile (A) and non‐pulsatile (B) circuits. [Color figure can be viewed at wileyonlinelibrary.com]

The test system consisted of an in vitro mock loop mimicking the left heart as well as an ECMO circuit (Figure 1). The mock loop consisted of a PMMA chamber representing the left ventricle (LV) which was connected via a 12mm Hancock bioprosthetic valved conduit (Medtronic, Minneapolis, MN) to a compliance chamber. This chamber reproduced the compliance of the systemic arterial vascular tree which could be tuned by adjusting the air volume in the compliance chamber. After passing through a hydraulic resistance element (modeling the systemic resistance), the fluid was returned to an open PMMA reservoir representing the atrium. The atrium was connected to the LV via a bi‐leaflet mechanical heart valve. The flow was driven by a computer‐controlled piston pump connected to the LV. The pump, the compliance and the resistance elements were tuned to obtain the desired pediatric flow rate and pressure.

For comparison, the experiments were also performed in non‐pulsatile conditions. To this end, the piston pump and the valve connecting the atrium to the ventricle were replaced by a roller pump (ISM404 Pump, Ismatec, Switzerland) directly connecting the atrium to the LV.

The cardiac output was set to 1 L/min for the pulsatile and non‐pulsatile configurations. The pulsatile experiments used a heart rate of 120 bpm with a systolic phase of 35% of the cardiac cycle and peak‐to‐peak aortic pressure of 40‐50 mmHg.^19^, ^20^

### 2.2 ECMO circuit

The ECMO circuit consisted of an Hilite 800 LT hollow fiber oxygenator (MEDOS Medizintechnik AG, Heilbronn, Germany), a Deltastream MDC DP3 diagonal pump (MEDOS Medizintechnik AG, Heilbronn, Germany), and ¼″ ID × 150 cm standardized length arterial/venous tubing with cannula pressure transducers. Four arterial cannulae (A: 8Fr, 10Fr, 12Fr, 14Fr; Medtronic, Inc., Minneapolis, MN, USA) were tested in combination with five venous cannulae (V: 12Fr, 14Fr, 16Fr, 18Fr, 20Fr; Medtronic, Inc., Minneapolis, MN, USA) for a total of 12 combinations: 8A‐12V, 8A‐14V, 8A‐16V, 10A‐12V, 10A‐14V, 10A‐16V, 12A‐14V, 12A‐16V, 12A‐18V, 12A‐20V, 14A‐18V, 14A‐20V. These cannulae had only holes at the tip and no side holes. The arterial cannula was inserted through a sealed connector into the compliance chamber, while the venous cannula was freely immersed into the open atrium (Figure 1). Once ECMO and mock loop were connected, the system was primed with blood‐mimicking fluid consisting of a 40% glycerin and 60% water mixture. Experiments were performed at a controlled temperature of 23°C to match the fluid viscosity of 3.7 × 10^−3^ Pa s of blood.

### 2.3 Flow and pressure measurement

The flow in the two circuits (ECMO and systemic mock loop) was measured with two transit‐time ultrasonic flow probes (TS410/ME‐11PXL, Transonic Systems, Ithaca, NY) located between the compliance chamber and the atrium for *Q*_Loop_ in the mock loop, and immediately downstream of the venous cannula the ECMO flow rate *Q*_ECMO_. Ventricular and aortic pressures, *p*_LV_ and *p*_Ao_, were recorded with two PBMN flush (Baumer, Frauenfeld, Switzerland) connected to the LV and the compliance chamber, respectively. Additional pressure sensors (Codan, Xtrans, Baar, Switzerland) were connected upstream of the arterial cannula (*p*_aC_), downstream of the oxygenator (*p*_oxy_), downstream of the venous cannula (*p*_vC_), in the pump (*p*_p_) and in the atrial chamber (*p*_LA_). Real‐time data from these sensors were acquired with PowerLab DAQ (ADInstruments, Dunedin, New Zealand) at 1 kHz. Prior to the measurements, the pressure value for the arterial cannula (*P*_aC_) was adjusted by subtracting the hydrostatic pressure offset due to the height difference relative to the aortic pressure sensor (*P*_Ao_), which was placed at the level of the aortic valve. The same adjustments were made for the venous cannula (*P*_vC_) with respect to the atrial pressure sensor (*P*_atrium_), located at the bottom of the atrial chamber.

### 2.4 Experimental protocol

For each cannula combination, the mock loop was tuned to a specific hemodynamic configuration while the ECMO circuit was disconnected by clamping both venous and arterial lines. Recordings started when the prescribed values for CO and aortic pressure were established. At this point, the clamps were removed and the speed of the ECMO pump (measured in revolutions per minute, RPM) was increased until an ECMO flow rate of 0.2 L/min was reached. After waiting for the parameters to stabilize (approximately 60 s), data were recorded. After that, the ECMO flow rate *Q*_ECMO_ was further increased by an increment of 0.2 L/min. This iterative procedure was repeated until a maximum pump speed of 8 500 RPM was reached. Higher pump speeds were not tested to avoid pump failure. After the maximum pump speed was reached, the ECMO pump was stopped, and the lines were clamped again. Before changing the cannulae combination and re‐connecting the ECMO, a second set of measurements was performed to ensure that the flow conditions had remained stable throughout the experiment. This routine was repeated three times for each cannula combination.

Data analysis was performed in Matlab (Mathworks, Natick, MA, USA) over 60 consecutive heartbeats for experiments with pulsatility and over 60 s for non‐pulsatile flow.

### 2.5 Hemodynamic parameters

The mean pressure drops across the arterial cannula and the venous cannula were calculated as

(1) $$∆P_{\text{Arterial},j}={\left\langle p_{\mathrm{aC}}-p_{\mathrm{Ao}}\right\rangle }_{j}$$

(2) $$∆P_{\text{Venous},j}={\left\langle p_{\mathrm{vC}}-p_{\text{atrium}}\right\rangle }_{j}$$

where $<.{>}_{j}$ is the temporal average over the *j* ^th^ pulse of period *T* with $j\in \left[1,60\right]$. The period *T* is equal to the length of a cardiac cycle for pulsatile flow and to the duration of a single pump revolution for non‐pulsatile flow. The total hemodynamic energy (*THE*) was computed for the arterial cannula from flow (*Q*_ECMO_) and pressure (*p*_aC_)^17^:

(3) $$\text{THE}=13320\;\frac{{\left\langle Q_{\text{ECMO}}∙p_{\mathrm{aC}}\right\rangle }_{j}}{{\left\langle Q_{\text{ECMO}}\right\rangle }_{j}\;}$$

The factor 13 320 is used to convert the equation from mmHg to J/m^3^. The *M‐number* was calculated using Montoya's equation^21^ as an indicator of the hemodynamic performance of vascular access devices:

(4) $$M=\mathrm{Log}\;\left(7.161∙10^{6}\;\mu^{-0.25}∆P\;{Q_{\text{ECMO}}}^{-1.75}\right)$$

where *μ* is the dynamic fluid viscosity (given in Poise) and ∆P the pressure drop at the arterial or venous cannula. In this formula, ∆P and *Q*_ECMO_ are given in mmHg and cm^3^/min, respectively. A cannula with a higher *M‐number* exhibits higher pressure drop at a given flow rate.

### 2.6 Statistical analysis

General linear mixed models (GLMM) were fitted to the quantitative results to compare pulsatile and non‐pulsatile conditions for each flow rate and cannulae combination.^22^ GLMM extends traditional linear regression to account for the within‐subject variability inherent in repeated measures designs and for data collected and summarized in groups. The model accounted for the repeated measurements within each experiment (cannulae combination and *Q*_ECMO_) and random effects for repeated measures on the same subject (variations in heartbeat for pulsatile flow and pump speed for non‐pulsatile flow).

## 3 RESULTS

### 3.1 Flow rate

**FIGURE 2:**
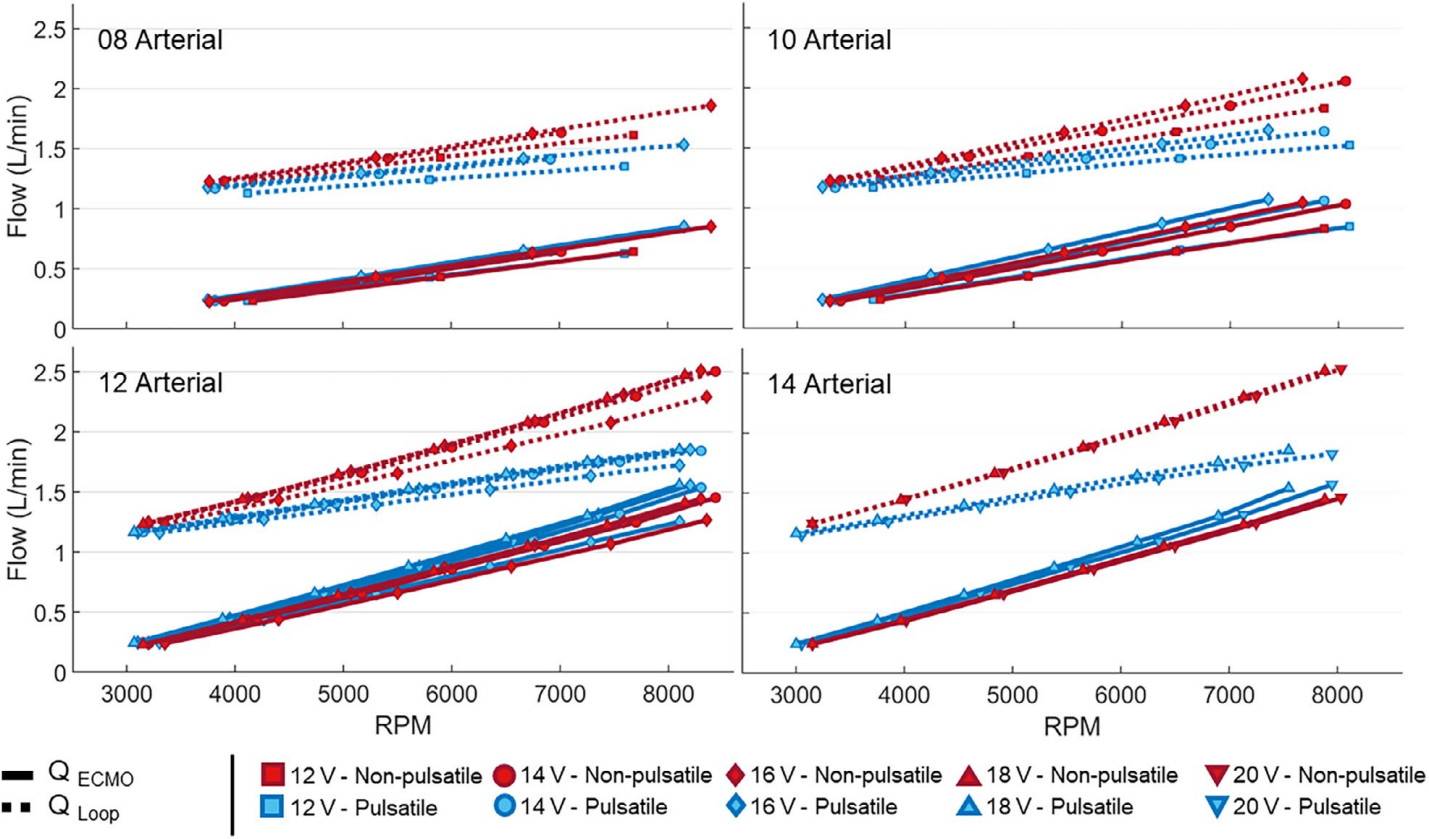
The ECMO (continuous line) and mock loop (dotted line) flow rates at different pump speeds for pulsatile (blue) and non‐pulsatile (red) flow conditions. The plots are grouped per arterial cannula size (*p* < 0.01 for *Q*_ECMO_ in all cannulae combinations). [Color figure can be viewed at wileyonlinelibrary.com]

Figure 2 shows the mean values for *Q*_Loop_ and *Q*_ECMO_ averaged over 60 heartbeats (pulsatile) or 60 s (non‐pulsatile). ECMO and mock loop flow rates increased linearly with increasing pump speed at pulsatile and non‐pulsatile flow conditions and for all cannulae combinations. However, the increase in *Q*_Loop_ was slower for pulsatile than for non‐pulsatile conditions. Larger arterial cannula size led to higher flow rates, whereas the venous cannula size had only a small effect on the flow rates. For the 14A cannula at peak pump speed, *Q*_Loop_ increased by 148% (non‐pulsatile) and by 80% (pulsatile) when compared to *Q*_Loop_ with disconnected ECMO.

### 3.2 Pressure

**FIGURE 3:**
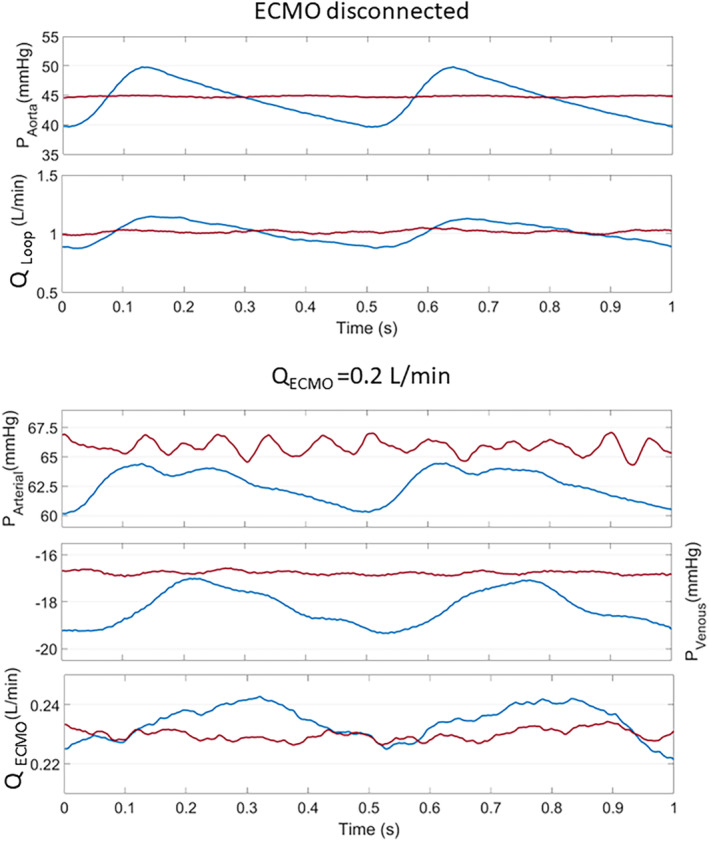
ECMO and flow loop pressures and flow rates recorded for pulsatile (blue) and non‐pulsatile (red) flow conditions. [Color figure can be viewed at wileyonlinelibrary.com]

**FIGURE 4:**
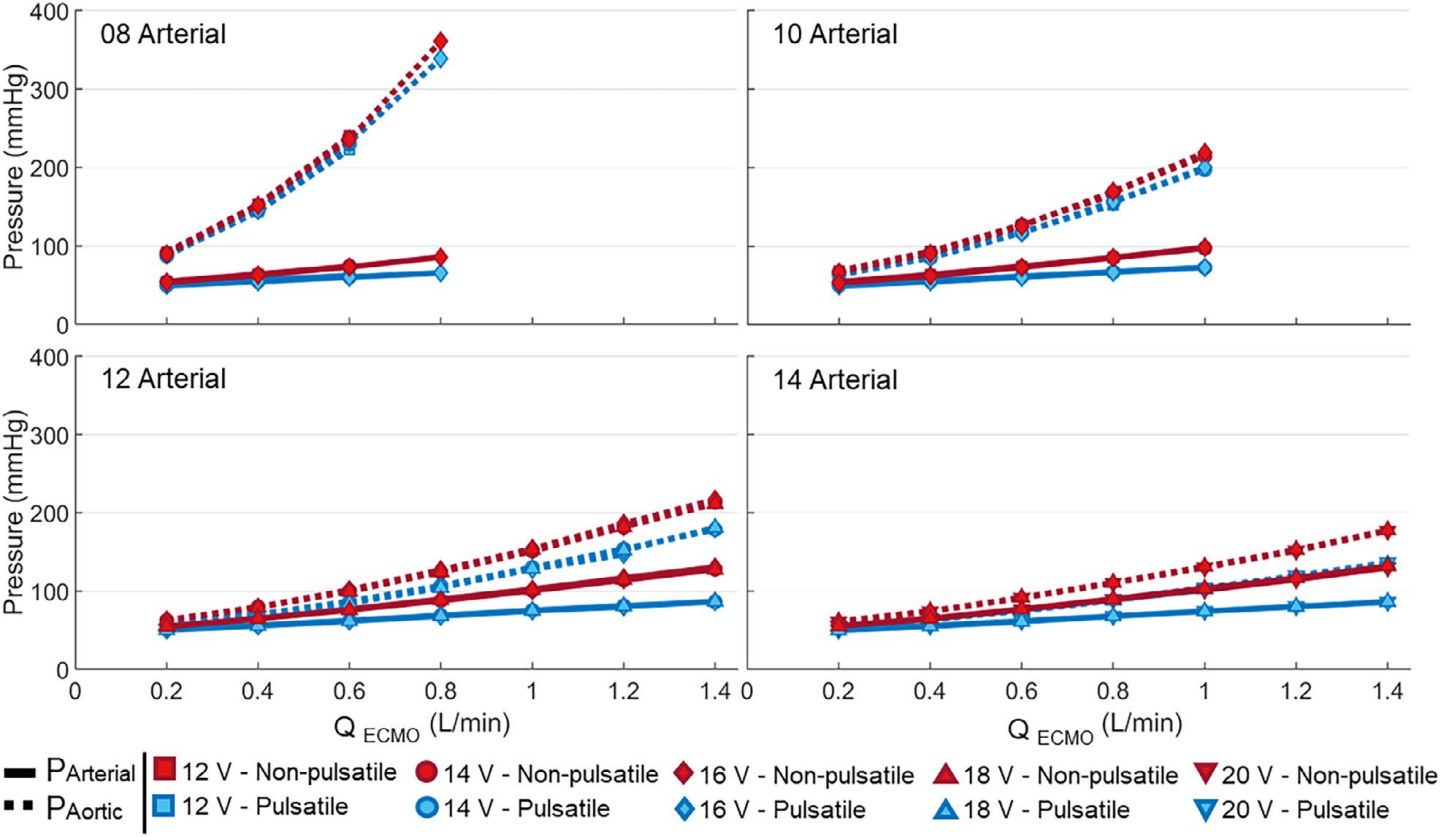
The arterial (continuous line) and aortic cannula (dotted line) pressure at different ECMO flow rates for pulsatile (blue) and non‐pulsatile (red) conditions. The plots are grouped per arterial cannula size (*p* < 0.01 for all cannulae combinations). [Color figure can be viewed at wileyonlinelibrary.com]

Under pulsatile conditions, pressure (and flow) profiles showed pulsatile characteristics across the ECMO in all tested combinations (Figure 3). Pulsatility also led to lower aortic and arterial pressures, *p*_Ao_ and *p*_LV_, for all cannulae combinations and flow rates (Figure 4). These pressures increased for increasing pump speed and decreasing arterial cannula size but did not depend on venous cannula size. The pressure differences between pulsatile and non‐pulsatile conditions were higher for larger arterial cannula sizes and increased for higher flow rates. For *Q*_ECMO_ = 0.2 L/min, the aortic and arterial pressure difference *p*_Ao_‐*p*_LA_ was <12% in all cannula combinations and increased to 34% (45.5 mmHg) and 25% (43.7 mmHg), respectively, for 14A‐20V combination at peak *Q*_ECMO_. The atrial pressure *p*_LA_ decreased with increasing flow rates for both non‐pulsatile and pulsatile flow conditions. This pressure reduction was higher for non‐pulsatile flow. At peak pump speed, the decrease in atrial pressure for pulsatile and non‐pulsatile conditions was 12% and 17%, respectively, for the cannula combination 08A‐12V. For the combination 14A‐20V, they were 16% and 30%.

### 3.3 Cannulae pressure drop

**FIGURE 5:**
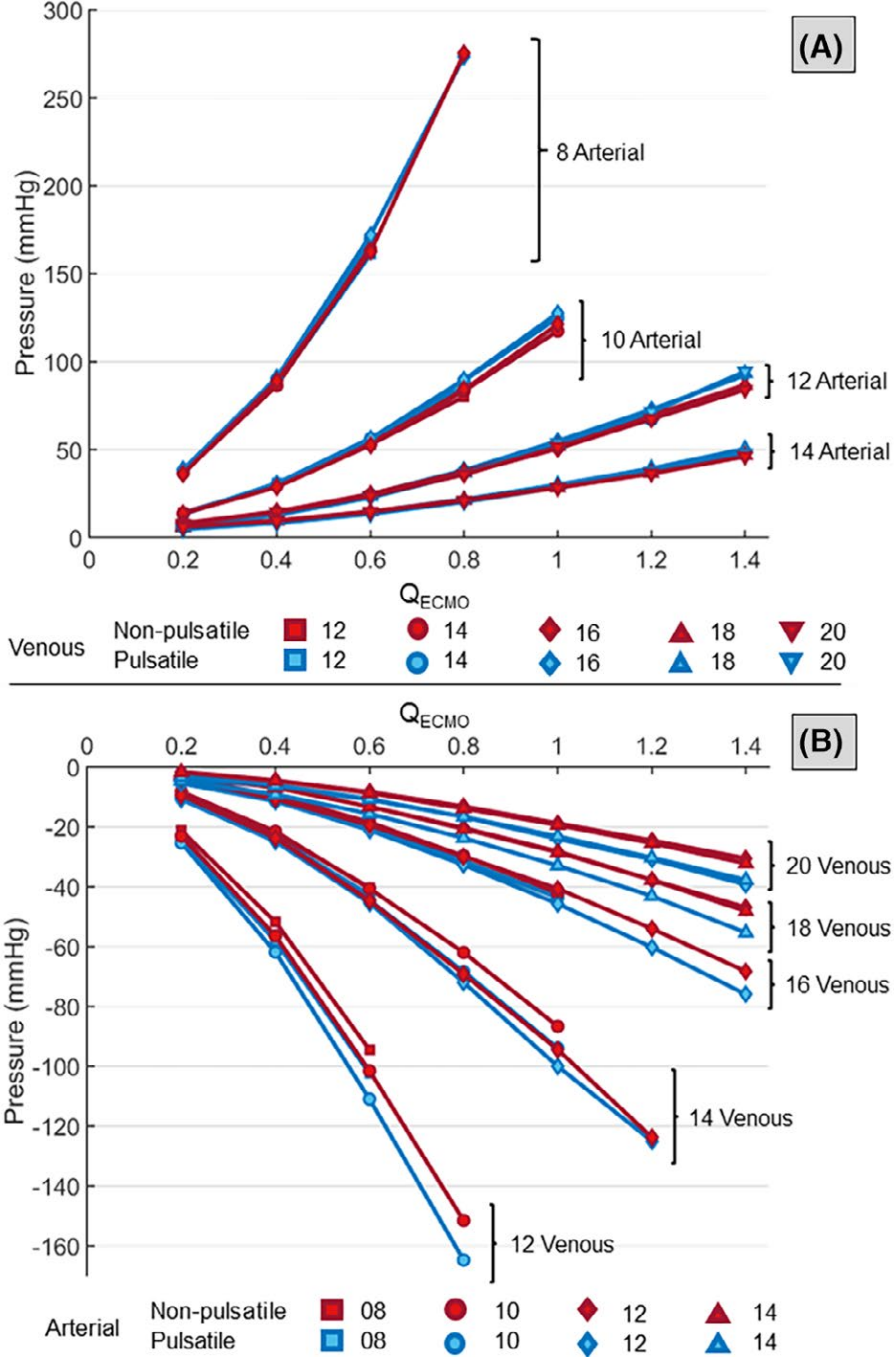
(A) The arterial cannula pressure drops at different flow rates for pulsatile (blue) and non‐pulsatile (red) conditions. (B) The venous cannula pressure loss at different flow rates for pulsatile (blue) and non‐pulsatile (red) conditions (*p* > 0.01 for arterial pressure drops combinations of 10A‐16V (0.2 L/min); *p* < 0.01 for the remaining arterial and venous pressure loss combinations). [Color figure can be viewed at wileyonlinelibrary.com]

Arterial and venous cannula pressure drops at different ECMO flow rates are shown in Figure 5. For increasing *Q*_ECMO_, the arterial cannula pressure drop increased. Similarly, the absolute value of the venous cannula pressure loss increased with *Q*_ECMO_ and decreasing venous size. However, the pressure loss depended only little on the arterial cannula size. In most combinations, pulsatile conditions resulted in higher absolute pressure loss on the venous side at peak pump speed.

### 3.4 M‐number

**FIGURE 6:**
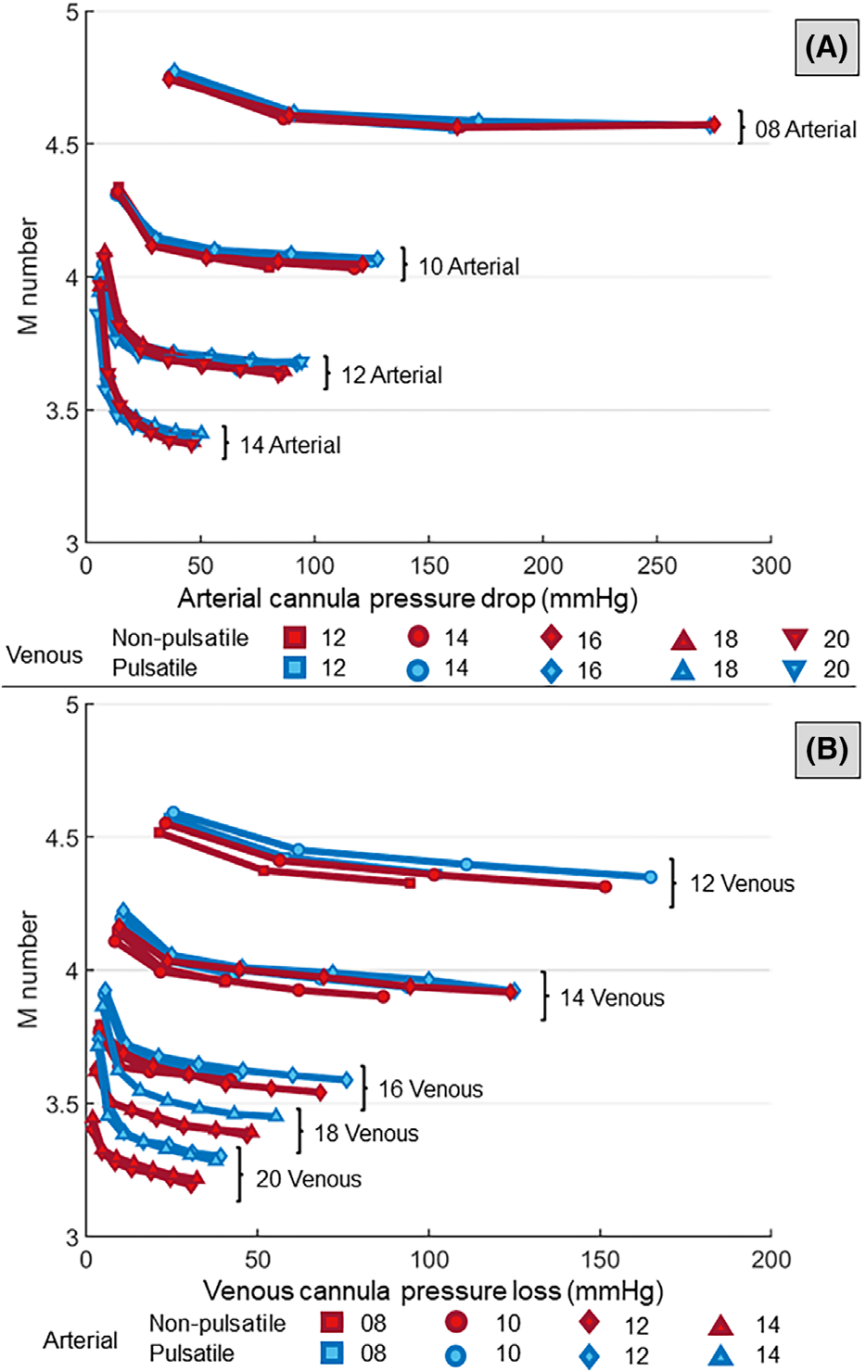
The *M‐number* computed for the arterial cannula (A) and the venous cannula (B) at different flow rates and cannulae combination for pulsatile (blue) and non‐pulsatile (red) conditions (*p* > 0.01 for cannulae combination of 12A‐16V (0.4 L/min and 0.8 L/min); *p* < 0.01 for the remaining cannulae combination). [Color figure can be viewed at wileyonlinelibrary.com]

The *M‐numbers* of all cannulae decreased with increasing cannula size and ECMO flow rate *Q*_ECMO_ (Figure 6). For the arterial cannula, no clear difference between the pulsatile and non‐pulsatile flow conditions was observed. For the venous cannula, differences between pulsatile and non‐pulsatile flow conditions increased for increasing venous size but remained less than 5%. Pulsatile flow showed generally higher *M* values for all cannula combinations.

### 3.5 Energy loss

**FIGURE 7:**
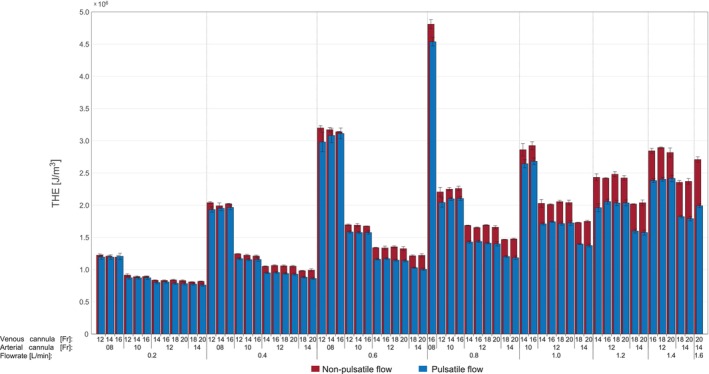
*THE* levels in the arterial cannula for different cannulae combinations at different flow rates (*p* < 0.01 for all configurations). [Color figure can be viewed at wileyonlinelibrary.com]

The arterial *THE* increased with increasing pump velocities and decreasing cannula size (Figure 7). The influence of the venous cannula size was very small in either pulsatile or non‐pulsatile conditions. The total hemodynamic energy was generally lower for pulsatile flow. The difference between pulsatile and non‐pulsatile flow increased with larger arterial cannula size. Differences between the two flow conditions increased with increasing flow rate. The results for non‐pulsatile flow conditions at peak pump speed in the 14Fr arterial cannula showed increased values reaching +47% compared to the respective value computed for the pulsatile flow. For the 12Fr and 10Fr arterial cannula, *THE* was +19% and +8% higher in non‐pulsatile conditions, respectively.

## 4 DISCUSSION

In ECMO, each component of the circuit should be carefully selected to reduce adverse effects such as hemolysis and thromboembolism. This selection must find the balance between providing sufficient metabolic support for the patient and limiting cell damage and hemolysis. When evaluating the performance of ECMO components, it is important to carefully simulate the hemodynamic conditions observed in the clinical setting. Blood‐related parameters, such as blood temperature and hematocrit, have been demonstrated to influence the results^23^, ^24^ and were considered in recent studies.^2^, ^6^, ^7^, ^8^ Wickramarachchi et al. recently introduced a mock loop mimicking resistance and pulsatility which was used to investigate cannulae performances in an adult ECMO circuit.^18^ However, to the authors' best knowledge, hemodynamic evaluations of ECMO cannulae under conditions that simulate the supported patient, have not been performed so far in a pediatric model.

In this study, physiological pressure and flow conditions were successfully obtained at the inlet/outlet of both cannulae. Moreover, it was possible to monitor and evaluate changes in the pressure upstream of the venous line. This parameter enables the evaluation of the suction of the venous cannula, which can lead to the collapse of the venous vascular site and subsequently severe instability and collapse of the ECMO circuit and support.

Under pulsatile test conditions, pulsatile flow and pressure were observed throughout the ECMO circuit while the flow was non‐pulsatile when a roller pump was used to drive the mock loop.

For non‐pulsatile conditions, the ECMO pump required a maximum of +5% higher pump speeds to reach a given ECMO flow rate as the resistance to the pump was higher. In case of pulsatile flow, the flow rate in the mock loop, *Q*_Loop_, did not increase at the same rate as the ECMO flow rate *Q*_ECMO_ (Figure 2). When *Q*_ECMO_ increases, also the pressure downstream the aorta increases, leading to an increased afterload to the valves which affects their opening and closing and may increase valvular regurgitation. A similar effect is expected when supporting the patient, as the afterload for the left side of the heart is increased due to the flow coming from the arterial cannula and the left valves are operating in a non‐physiological condition.^25^, ^26^ For non‐pulsatile flow, we do not see this phenomenon because the used roller pump creates a constant flow rate by design.

The response to increasing flow rate observed in the non‐pulsatile pseudo‐patient showed a higher increase in the aortic and arterial pressure in both the ECMO and the mock loop. However, the pressure drops at both cannulae ($∆P_{\text{Arterial}}\;\text{and}∆P_{\text{Venous}}$, Figure 5) were unaffected and comparable among the two pumping systems.

The total hemodynamic energy was lower for pulsatile flows, which was unexpected as cyclic velocity variations are associated with higher kinetic energy.^7^, ^27^ However, the total hemodynamic pressure not only accounts for the kinetic energy carried by the blood but also for its potential energy. The potential energy is associated with the pressure exerted on the cannulae wall, which were significantly higher for in case of non‐pulsatile flow and compensated for decreases in kinetic energy.

However, parameters based on differential values such as *M‐number* did not show significant differences between the two flow conditions. This can be attributed to the overall pressure increase in the whole circuit for non‐pulsatile conditions. Therefore, differential quantities like the pressure drop at the cannula remain similar between pulsatile and non‐pulsatile conditions. In fact, the hydraulic resistance of a conduit is independent of the pressure at its inlet.

When testing the system in a non‐pulsatile condition, the flow in the circuit is subject to a transient. In this phase, the air in the compliance chamber is more and more compressed, with a consequent loss of its compliance. As a volumetric pump determines the flow rate in the mock patient, the pressure increases until the compliance is saturated to maintain the flow rate set as baseflow in the mock patient, leading to pressure values above physiological thresholds. This intrinsic mechanism characterizing studies under non‐pulsatile conditions limits the use of compliant elements while the design flow driving mechanism needs to be carefully designed as it might lead to overestimation of the systemic pressures and ECMO support despite the realistic values of pressure gradient.

To conclude, we demonstrate that patient pulsatility is maintained in the ECMO circuit and plays a role in the evaluation of the device. Under pulsatile flow, the levels of *THE* are lower than under non‐pulsatile flow. Thus, we suggest that the testing of different ECMO components should also be performed under pulsatile conditions to avoid misleading results.

## 5 CONCLUSION

This study presents a test bench that considers systemic compliance and resistance of a toddler. The mock patient was used to test ECMO cannulae under and pulsatile versus non‐pulsatile flow conditions. Our findings highlight the necessity of considering pulsatile flow in ECMO testing to avoid discrepancies between in vitro specifications and clinical performance.

The pressure increases in the system were significantly higher for non‐pulsatile conditions. While this variation did not affect differential parameters such as *M‐number* and pressure drops, afterloads, and total hemodynamic energy were affected. The higher afterload associated with increasing cardiac output provided by ECMO resulted in lower patient support under pulsatile conditions compared to non‐pulsatile conditions. Although higher levels of kinetic energy were expected for pulsatile flows, the *THE* recorded in this configuration was lower for pulsatile conditions because higher pressure led to higher potential energy. These effects were more pronounced with larger arterial cannulae and at higher pump speeds, with little dependency on the venous size.

To conclude, we demonstrate that patient pulsatility is maintained in the ECMO circuit and plays a role in the evaluation of the device. The pulsatile testing conditions allowed for a better model of the real‐world clinical scenario, where the patient's aortic valve opens and closes, preserving the effect of vascular compliance. Under non‐pulsatile conditions, results showed an overestimation of the support to the patient. However, clinically used cannulae combinations can still provide the required flow for different weight categories of pediatric patients. By realizing a physiological ECLS circuit and demonstrating the influence of patient pulsatility in ECMO evaluation, this study provided a wide range of compelling data and a scientific basis for making cannula selection in this challenging patient group, where even small differences in the supported flow can be lifesaving. Therefore, it is essential that different ECMO components be tested under pulsatile conditions, as in the real clinical setting.

## 6 LIMITATIONS

Despite the more realistic testing conditions introduced using a mock patient for ECMO cannula evaluations, precise geometry and mechanical properties of the vascular access of the cannulae were not accounted for in the study. Such features could contribute to the dynamics of the flow patterns in the mixing zones near the cannulae, possibly leading to higher flow rate due to the rigid chambers and tubing.^18^, ^28^ However, the mock patient replicated physiological pediatric pressure and flow within the circuit enabling a systematic comparison of different cannulae settings under both pulsatile and non‐pulsatile flow conditions.

Cellular blood components were not included in the blood mimicking solution, and therefore, it was not possible to observe hemolytic effects, shear thinning, and other biological factors.

The study did not replicate specific pathological or physiological conditions and the mock loop aimed to mimic an idealized patient. The acquired pathology as well as physiological conditions can lead to valvular regurgitation, reduced cardiac contractility or other variables in the patient which need to be analyzed separately.

## AUTHOR CONTRIBUTIONS

Lorenzo Ferrari: Conceptualization (equal); data curation; formal analysis (equal); investigation (equal); methodology (equal); visualization; writing – original draft (equal); review and editing (equal). Maris Bartkevics: Conceptualization (equal); formal analysis (support); methodology (equal); writing – review and editing (equal). Hansjörg Jenni: Conceptualization (support); formal analysis (support); methodology (equal); writing – review and editing (equal). Alexander Kadner: Formal analysis (supporting); supervision (equal); writing – review and editing (equal). Matthias Siepe: Formal analysis (supporting); supervision (equal); funding acquisition (equal); writing – review and editing (equal). Dominik Obrist: Conceptualization (equal); formal analysis (supporting); funding acquisition (equal); supervision (equal) – review and editing (equal)

## CONFLICT OF INTEREST STATEMENT

The authors declare that they have no conflicts of interest with the contents of this article.
